# 3,7,11-Tris{4-[(1*R*,3*S*,4*S*)-neomenth­yl­oxy]phen­yl}tri[1,2,4]triazolo[4,3-*a*:4′,3′-*c*:4′′,3′′-*e*][1,3,5]triazine–chloro­form–ethanol (1/1/1)

**DOI:** 10.1107/S1600536813003498

**Published:** 2013-02-09

**Authors:** Karoline Herget, Dieter Schollmeyer, Heiner Detert

**Affiliations:** a University Mainz, Institut of Organic Chemistry, Duesbergweg 10-14, 55099 Mainz, Germany

## Abstract

The title compound, C_54_H_69_N_9_O_3_·CHCl_3_·C_2_H_5_OH, was prepared by a threefold nucleophilic substitution of *p*-neomenthyloxyphenyl­tetra­zole on cyanuric chloride followed by threefold cyclo­elimination of nitro­gen and ring closure. The central tris­triazolotriazine is roughly planar with a maximum deviation of 0.089 (7) Å but the adjacent benzene rings are twisted out of this plane. N—C—C—C torsion angles of −80.2 (9), 159.3 (7) and 50.6 (10)° destroy the formal *C*3 symmetry. Cavities are found between the phen­oxy residues: one is occupied by a chloro­form mol­ecule, another by ethanol forming a hydrogen bond to a triazole ring while two isopropyl groups point into the third void. One methyl group and the chloro­frm mol­ecule are disorderd and were refined using a split model.

## Related literature

For the synthesis of related tris­triazolotriazines, see: Hofmann & Erhardt (1912 ▶); Huisgen *et al.* (1961 ▶); Glang *et al.* (2008 ▶). For structures of tetra­zoles, see: Rieth *et al.* (2011 ▶). For structures of related tris­triazolotriazines, see: Christiano *et al.* (2008 ▶). For liquid-crystalline tris­triazolotriazines, see: Christiano *et al.* (2008 ▶, 2012 ▶); Glang *et al.* (2013 ▶). For isomeric tris­triazolotriazines, see: Tartakovsky *et al.* (2005 ▶); Borchmann *et al.* (2010 ▶). For star-shaped conjugated oligomers, see: Schmitt *et al.* (2011 ▶); Detert *et al.* (2010 ▶). 
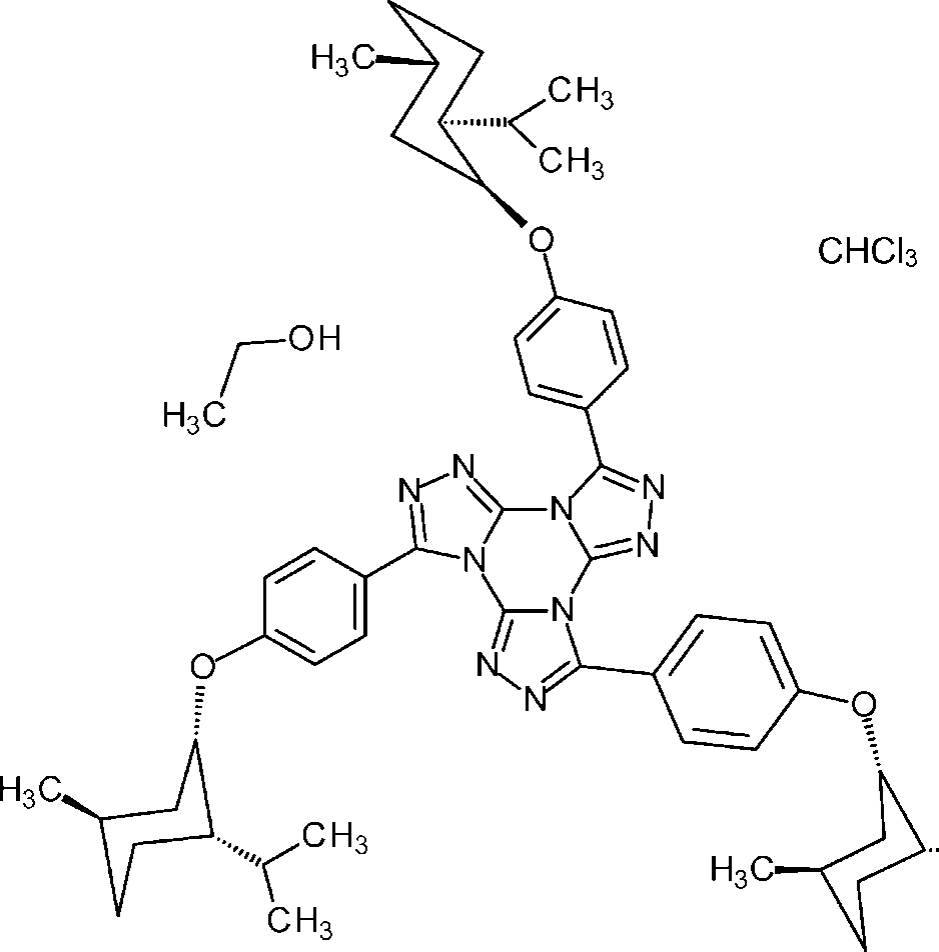


## Experimental

### 

#### Crystal data

C_54_H_69_N_9_O_3_·CHCl_3_·C_2_H_6_O
*M* *_r_* = 1057.62
Orthorhombic, 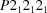

*a* = 6.6562 (5) Å
*b* = 14.1000 (14) Å
*c* = 60.756 (5) Å
*V* = 5702.1 (8) Å^3^
*Z* = 4
Mo *K*α radiation
μ = 0.21 mm^−1^
*T* = 193 K
0.5 × 0.06 × 0.02 mm

#### Data collection

Stoe IPDS 2T diffractometer
32105 measured reflections
10791 independent reflections
2815 reflections with *I* > 2σ(*I*)
*R*_int_ = 0.168

#### Refinement

*R*[*F* ^2^ > 2σ(*F* ^2^)] = 0.061
*wR*(*F* ^2^) = 0.153
*S* = 0.78
10791 reflections
687 parameters
26 restraints
H-atom parameters constrained
Δρ_max_ = 0.31 e Å^−3^
Δρ_min_ = −0.25 e Å^−3^
Absolute structure: Flack (1983 ▶), 4585 Friedel pairs
Flack parameter: 0.03 (14)

### 

Data collection: *X-AREA* (Stoe & Cie, 2011 ▶); cell refinement: *X-AREA*; data reduction: *X-RED* (Stoe & Cie, 2011 ▶); program(s) used to solve structure: *SIR97* (Altomare *et al.*, 1999 ▶); program(s) used to refine structure: *SHELXL97* (Sheldrick, 2008 ▶); molecular graphics: *PLATON* (Spek, 2009 ▶); software used to prepare material for publication: *PLATON*.

## Supplementary Material

Crystal structure: contains datablock(s) I, global. DOI: 10.1107/S1600536813003498/nc2304sup1.cif

Structure factors: contains datablock(s) I. DOI: 10.1107/S1600536813003498/nc2304Isup2.hkl

Additional supplementary materials:  crystallographic information; 3D view; checkCIF report

## Figures and Tables

**Table 1:** Hydrogen-bond geometry (Å, °)

*D*—H⋯*A*	*D*—H	H⋯*A*	*D*⋯*A*	*D*—H⋯*A*
O2*L*—H2*L*⋯N12^i^	0.84	2.11	2.896 (10)	156

Symmetry code: (i) 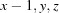.

**Fig. 1.**
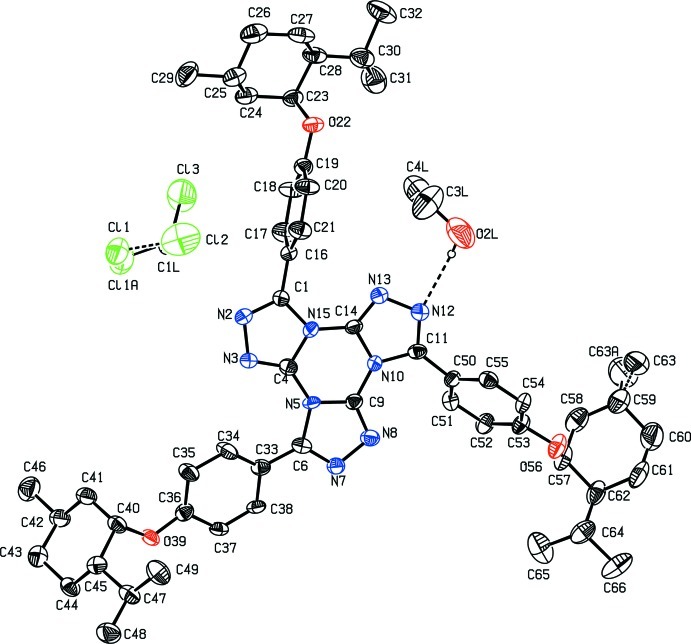
Crystal structure of the title compound with labeling and displacement ellipsoids drawn at the 30% probability level.

## supplementary crystallographic information

### Comment

As part of a larger project on discotic molecules for optical and LC applications (Schmitt *et al.* 2011; Borchmann *et al.* 2010) we prepared the title compound as a chiral dopant for columnar LC phases of tristriazolotriazines (Christiano *et al.* 2008, Glang *et al.* 2013). The central tristriazolotriazine system is essentially planar with a maximal deviation of 0.089 (7) Å at C1 from the mean plane. The three benzene ring are twisted out of this plane, the torsion angles are -80.2 (9)° (N2—C1—C16—C17), 159.3 (7)° (N7—C6—C33—C34), and 50.6 (10)° (N12—C11—C50—C55).

The voluminous neomethyl residues inhibit a close packing of the tristriazolotriazines, the empty space is filled in part with solvent molecules. A disordered chloroform molecule and an ethanol molecule flank the orthogonal benzene ring, the alcohol forms a hydrogen bridge (O2l—H2l···N12) to N12 of the triazole.

All neomethyl units adopt conformations with equatorial methyl and isopropyl groups. Like the torsion of the benzene rings, the orientation of the neomenthyl goups break the possible C3 symmetry: Two isopropyl groups point into the empty space, the third one is oriented to the space occupied with the smaller solvent molecule.

### Experimental

3,7,11-Tris-(4-(1*R*,3*S*,4*S*)-neomenthyloxyphenyl)tris([1,2,4] triazolo)[4,3 - a:4',3'-c:4'',3''-e][1,3,5]triazine: 5-(4-Neomenthyloxyphenyl)tetrazole was prepared by a Mitsunobu-reaction of *p*-cyanophenol and (-)-menthol (53%) followed by an addition of hydrazoic acid (NaN_3_, Et_3_NHCl, toluene, 87%, mp: 513 K), and recrystallization from ethanol/toluene. Collidine (1 ml) was added to a stirred suspension of the tetrazole (500 mg, 1.66 mmol) and cyanuric chloride (93 mg, 0.5 mmol) in xylenes. After stirring for 16 h, the mixture was slowly heated to 343 K (4 h). Hydrochloric acid (2 N, 10 ml) was added and the organic layer diluted with ethyl acetate (100 ml). The organic solution was washed with water and brine, dried, and concentrated. Purification by column chromatography (basic aluminium oxide, toluene/ethyl acetate = 19/1 (*v*/*v*)). Yield: 213 mg (70%) of an off-white solid with m.p. = 484–485 K. Single crystals were obtained from slow crystallization from chloroform/ethanol

### Refinement

Hydrogen atoms attached to carbons and oxygen were placed at calculated positions (methyl H atoms allowed to rotate but not to tip) with C—H = 0.93 Å for aromatic, 0.97 Å for methylene, 0.96 Å for methyl H atoms and 0.84 Å for O—H distance and were refined in the riding-model approximation with a common isotropic displacement parameters for those H atoms connected to the same C atom. One methyl group (C63) and the trichloromethan molecule are disorderd. The C62–C63/C63*a* distances were refined assuming standard C–C distances (1.54 (2) Å). Thermal parameters for C63,C63*a* and the solvent CHCl_3_ molecule are restrained to isotropic manner. The absolute structure was determined on the basis of 4585 Friedel pairs.

### Crystal data

**Table:**

C_54_H_69_N_9_O_3_·CHCl_3_·C_2_H_6_O	*D*_x_ = 1.232 Mg m^−^^3^
*M**_r_* = 1057.62	Melting point: 422 K
Orthorhombic, *P*2_1_2_1_2_1_	Mo *K*α radiation, λ = 0.71073 Å
Hall symbol: P 2ac 2ab	Cell parameters from 8050 reflections
*a* = 6.6562 (5) Å	θ = 2.2–25.0°
*b* = 14.1000 (14) Å	µ = 0.21 mm^−^^1^
*c* = 60.756 (5) Å	*T* = 193 K
*V* = 5702.1 (8) Å^3^	Needle, colourless
*Z* = 4	0.5 × 0.06 × 0.02 mm
*F*(000) = 2256	

### Data collection

**Table:**

Stoe IPDS 2T diffractometer	2815 reflections with *I* > 2σ(*I*)
Radiation source: sealed Tube	*R*_int_ = 0.168
Graphite monochromator	θ_max_ = 25.8°, θ_min_ = 2.2°
Detector resolution: 6.67 pixels mm^-1^	*h* = −7→8
rotation method scans	*k* = −17→17
32105 measured reflections	*l* = −74→74
10791 independent reflections	

### Refinement

**Table:**

Refinement on *F*^2^	Secondary atom site location: difference Fourier map
Least-squares matrix: full	Hydrogen site location: inferred from neighbouring sites
*R*[*F*^2^ > 2σ(*F*^2^)] = 0.061	H-atom parameters constrained
*wR*(*F*^2^) = 0.153	*w* = 1/[σ^2^(*F*_o_^2^) + (0.0337*P*)^2^] where *P* = (*F*_o_^2^ + 2*F*_c_^2^)/3
*S* = 0.78	(Δ/σ)_max_ = 0.001
10791 reflections	Δρ_max_ = 0.31 e Å^−^^3^
687 parameters	Δρ_min_ = −0.25 e Å^−^^3^
26 restraints	Absolute structure: Flack (1983), 4585 Friedel pairs
Primary atom site location: structure-invariant direct methods	Flack parameter: 0.03 (14)

### Special details

**Table:**

Experimental. H-NMR (300 MHz, CDCl_3_): 8.10 ("d", 6 H, 2-H, 6-H, phenyl), 7.07 ("d", 6 H, 3-H, 5-H, phenyl), 4.76 (bs, 3 H) 2.17 (dq, 3 H, J = 15 Hz, J = 2.3 Hz), 1.84 - 1.67 (m, 15 H), 1.18 - 0.95 (m, 9 H), 0.95 (d, J = 8.1 Hz, 9 H), 0.88 (d, 9 H), 0.86 (d, 9 H).C-NMR (75 MHz, CDCl_3_): 161.4, 151.2, 140.6, 132.1, 115.4, 115.4, 73.7, 47.2, 37.7, 35.1, 29.4, 26.4, 25.0, 22.4, 21.2, 21.0.IR (ATR): 2945, 2924, 2868, 2845, 2350, 1592, 1530, 1480, 1457, 1427, 1369, 1290, 1252, 1223, 1200, 1176, 1150, 1121, 1025, 1012, 963, 940, 910, 892, 832, 730, 708.MS (FD): 1784 (1%) [*M*_2_]^+^; 892 (100%) [*M*]^+^; 755 (1%) [*M* - neomenthyl]Combustion analysis: calc. for C_54_H_69_N_9_O_3_: C: 72.70%, H: 7.80%, N: 14.13%. Found: C: 72.36%, H: 7.86%, N: 14.09%.
Geometry. All e.s.d.'s (except the e.s.d. in the dihedral angle between two l.s. planes) are estimated using the full covariance matrix. The cell e.s.d.'s are taken into account individually in the estimation of e.s.d.'s in distances, angles and torsion angles; correlations between e.s.d.'s in cell parameters are only used when they are defined by crystal symmetry. An approximate (isotropic) treatment of cell e.s.d.'s is used for estimating e.s.d.'s involving l.s. planes.
Refinement. Refinement of *F*^2^ against ALL reflections. The weighted *R*-factor *wR* and goodness of fit *S* are based on *F*^2^, conventional *R*-factors *R* are based on *F*, with *F* set to zero for negative *F*^2^. The threshold expression of *F*^2^ > σ(*F*^2^) is used only for calculating *R*-factors(gt) *etc*. and is not relevant to the choice of reflections for refinement. *R*-factors based on *F*^2^ are statistically about twice as large as those based on *F*, and *R*- factors based on ALL data will be even larger.

### Fractional atomic coordinates and isotropic or equivalent isotropic displacement parameters (Å^2^)

**Table:**

	*x*	*y*	*z*	*U*_iso_*/*U*_eq_	Occ. (<1)
C1	0.8472 (11)	0.8349 (5)	0.89333 (11)	0.0467 (18)	
N2	0.7047 (9)	0.8376 (4)	0.90835 (9)	0.0569 (17)	
N3	0.5733 (9)	0.7602 (4)	0.90507 (9)	0.0561 (16)	
C4	0.6395 (11)	0.7149 (5)	0.88820 (11)	0.0483 (19)	
N5	0.5718 (9)	0.6368 (3)	0.87701 (8)	0.0432 (14)	
C6	0.4029 (11)	0.5766 (5)	0.87885 (11)	0.0482 (18)	
N7	0.4046 (9)	0.5156 (4)	0.86296 (9)	0.0510 (16)	
N8	0.5717 (10)	0.5318 (4)	0.84955 (9)	0.0566 (16)	
C9	0.6616 (12)	0.6032 (5)	0.85831 (10)	0.0472 (19)	
N10	0.8375 (9)	0.6470 (4)	0.85141 (8)	0.0447 (15)	
N12	1.1267 (9)	0.6842 (4)	0.83695 (9)	0.0566 (17)	
N13	1.0943 (10)	0.7462 (4)	0.85493 (9)	0.0549 (16)	
C11	0.9779 (12)	0.6261 (5)	0.83499 (10)	0.049 (2)	
C14	0.9214 (12)	0.7215 (5)	0.86274 (11)	0.0473 (19)	
N15	0.8146 (9)	0.7560 (4)	0.88032 (8)	0.0453 (15)	
C16	0.9937 (12)	0.9092 (5)	0.88891 (11)	0.051 (2)	
C17	0.9390 (13)	0.9861 (6)	0.87639 (12)	0.079 (2)	
H17	0.8062	0.9897	0.8708	0.094*	
C18	1.0719 (13)	1.0574 (5)	0.87186 (12)	0.070 (2)	
H18	1.0307	1.1100	0.8632	0.084*	
C19	1.2640 (13)	1.0533 (6)	0.87984 (12)	0.057 (2)	
C20	1.3199 (12)	0.9785 (5)	0.89251 (12)	0.067 (2)	
H20	1.4525	0.9760	0.8983	0.080*	
C21	1.1854 (13)	0.9051 (5)	0.89720 (12)	0.068 (2)	
H21	1.2262	0.8529	0.9060	0.081*	
O22	1.4113 (7)	1.1184 (3)	0.87552 (7)	0.0593 (13)	
C23	1.3491 (12)	1.2162 (5)	0.87097 (12)	0.059 (2)	
H23	1.2488	1.2167	0.8587	0.071*	
C24	1.2581 (11)	1.2595 (5)	0.89107 (13)	0.068 (2)	
H24A	1.1383	1.2221	0.8953	0.082*	
H24B	1.2123	1.3245	0.8874	0.082*	
C25	1.3987 (14)	1.2647 (5)	0.91061 (13)	0.074 (2)	
H25	1.4396	1.1989	0.9148	0.089*	
C26	1.5830 (15)	1.3194 (6)	0.90378 (16)	0.100 (3)	
H26A	1.6802	1.3205	0.9161	0.120*	
H26B	1.5449	1.3857	0.9004	0.120*	
C27	1.6812 (13)	1.2752 (6)	0.88377 (15)	0.084 (3)	
H27A	1.7299	1.2111	0.8877	0.101*	
H27B	1.7995	1.3139	0.8796	0.101*	
C28	1.5402 (12)	1.2670 (5)	0.86361 (15)	0.073 (2)	
H28	1.5007	1.3330	0.8594	0.087*	
C29	1.2996 (14)	1.3120 (6)	0.93033 (14)	0.110 (3)	
H29A	1.2566	1.3761	0.9263	0.164*	
H29B	1.3958	1.3155	0.9425	0.164*	
H29C	1.1825	1.2748	0.9349	0.164*	
C30	1.6331 (13)	1.2217 (6)	0.84330 (16)	0.085 (3)	
H30	1.6838	1.1574	0.8474	0.103*	
C31	1.4876 (15)	1.2106 (6)	0.82425 (13)	0.118 (4)	
H31A	1.4552	1.2732	0.8182	0.177*	
H31B	1.3642	1.1800	0.8295	0.177*	
H31C	1.5493	1.1714	0.8128	0.177*	
C32	1.8185 (14)	1.2838 (6)	0.83536 (16)	0.127 (4)	
H32A	1.9236	1.2827	0.8467	0.190*	
H32B	1.7746	1.3493	0.8329	0.190*	
H32C	1.8718	1.2576	0.8216	0.190*	
C33	0.2432 (12)	0.5763 (5)	0.89560 (11)	0.051 (2)	
C34	0.2663 (12)	0.6180 (6)	0.91587 (11)	0.073 (3)	
H34	0.3888	0.6494	0.9193	0.088*	
C35	0.1134 (12)	0.6154 (6)	0.93159 (11)	0.073 (3)	
H35	0.1299	0.6478	0.9452	0.087*	
C36	−0.0618 (13)	0.5657 (5)	0.92750 (11)	0.055 (2)	
C37	−0.0811 (11)	0.5221 (4)	0.90715 (11)	0.0486 (18)	
H37	−0.1997	0.4872	0.9040	0.058*	
C38	0.0668 (11)	0.5280 (4)	0.89134 (10)	0.0482 (19)	
H38	0.0474	0.4986	0.8774	0.058*	
O39	−0.2111 (7)	0.5509 (3)	0.94215 (7)	0.0571 (14)	
C40	−0.1781 (11)	0.5796 (5)	0.96488 (10)	0.058 (2)	
H40	−0.0324	0.5720	0.9685	0.070*	
C41	−0.2355 (12)	0.6816 (6)	0.96782 (11)	0.065 (2)	
H41A	−0.1635	0.7200	0.9567	0.078*	
H41B	−0.1899	0.7028	0.9825	0.078*	
C42	−0.4608 (13)	0.7012 (6)	0.96576 (12)	0.067 (2)	
H42	−0.5027	0.6829	0.9505	0.080*	
C43	−0.5725 (12)	0.6362 (5)	0.98192 (11)	0.068 (2)	
H43A	−0.7190	0.6468	0.9805	0.082*	
H43B	−0.5329	0.6525	0.9972	0.082*	
C44	−0.5252 (11)	0.5309 (5)	0.97753 (11)	0.062 (2)	
H44A	−0.5983	0.4910	0.9883	0.075*	
H44B	−0.5723	0.5136	0.9626	0.075*	
C45	−0.3010 (12)	0.5114 (5)	0.97933 (11)	0.058 (2)	
H45	−0.2624	0.5247	0.9949	0.070*	
C46	−0.5138 (15)	0.8043 (5)	0.96894 (12)	0.097 (3)	
H46A	−0.4526	0.8420	0.9572	0.145*	
H46B	−0.4631	0.8259	0.9832	0.145*	
H46C	−0.6601	0.8118	0.9685	0.145*	
C47	−0.2482 (12)	0.4076 (6)	0.97495 (12)	0.069 (2)	
H47	−0.2959	0.3918	0.9598	0.083*	
C48	−0.3554 (13)	0.3398 (5)	0.99113 (12)	0.092 (3)	
H48A	−0.3272	0.3596	1.0063	0.138*	
H48B	−0.3064	0.2750	0.9889	0.138*	
H48C	−0.5006	0.3419	0.9885	0.138*	
C49	−0.0199 (12)	0.3867 (6)	0.97584 (12)	0.095 (3)	
H49A	0.0019	0.3179	0.9756	0.142*	
H49B	0.0366	0.4131	0.9894	0.142*	
H49C	0.0463	0.4157	0.9631	0.142*	
C50	0.9513 (12)	0.5551 (5)	0.81750 (10)	0.0484 (19)	
C51	0.7809 (12)	0.5539 (5)	0.80502 (10)	0.055 (2)	
H51	0.6734	0.5955	0.8085	0.067*	
C52	0.7629 (12)	0.4924 (5)	0.78727 (11)	0.061 (2)	
H52	0.6447	0.4921	0.7785	0.074*	
C53	0.9192 (13)	0.4324 (5)	0.78265 (10)	0.058 (2)	
C54	1.0923 (12)	0.4314 (5)	0.79545 (10)	0.054 (2)	
H54	1.1981	0.3883	0.7923	0.065*	
C55	1.1079 (12)	0.4943 (5)	0.81291 (10)	0.057 (2)	
H55	1.2261	0.4954	0.8217	0.068*	
O56	0.9187 (8)	0.3675 (3)	0.76562 (8)	0.0770 (16)	
C57	0.7784 (13)	0.3828 (6)	0.74747 (12)	0.076 (3)	
H57	0.6440	0.4011	0.7535	0.091*	
C58	0.8550 (16)	0.4602 (7)	0.73252 (15)	0.096 (3)	
H58A	0.8685	0.5194	0.7412	0.116*	
H58B	0.7545	0.4717	0.7208	0.116*	
C59	1.056 (2)	0.4378 (8)	0.72183 (17)	0.124 (4)	
H59	1.1552	0.4296	0.7341	0.148*	0.60
H59A	1.1729	0.4424	0.7321	0.148*	0.40
C60	1.0396 (18)	0.3423 (9)	0.71027 (18)	0.146 (5)	
H60A	0.9473	0.3488	0.6976	0.175*	
H60B	1.1734	0.3246	0.7045	0.175*	
C61	0.9646 (15)	0.2633 (7)	0.72493 (16)	0.105 (4)	
H61A	1.0657	0.2505	0.7365	0.126*	
H61B	0.9490	0.2048	0.7161	0.126*	
C62	0.7627 (13)	0.2874 (7)	0.73586 (14)	0.083 (3)	
H62	0.6627	0.2953	0.7237	0.099*	
C63	1.146 (4)	0.5107 (17)	0.7059 (4)	0.131 (9)	0.60
H63A	1.1717	0.5702	0.7138	0.196*	0.60
H63B	1.0519	0.5224	0.6938	0.196*	0.60
H63C	1.2729	0.4863	0.7000	0.196*	0.60
C63A	1.041 (6)	0.523 (3)	0.7052 (7)	0.16 (2)	0.40
H63D	1.1742	0.5354	0.6989	0.246*	0.40
H63E	0.9927	0.5792	0.7129	0.246*	0.40
H63F	0.9475	0.5062	0.6933	0.246*	0.40
C64	0.6838 (16)	0.2073 (6)	0.75075 (14)	0.089 (3)	
H64	0.7844	0.1981	0.7628	0.107*	
C65	0.4891 (16)	0.2355 (8)	0.76140 (16)	0.145 (5)	
H65A	0.3960	0.2588	0.7501	0.217*	
H65B	0.5141	0.2857	0.7722	0.217*	
H65C	0.4300	0.1804	0.7688	0.217*	
C66	0.6701 (18)	0.1141 (6)	0.73813 (16)	0.144 (5)	
H66A	0.6110	0.1257	0.7236	0.216*	
H66B	0.5856	0.0694	0.7463	0.216*	
H66C	0.8050	0.0873	0.7364	0.216*	
C1L	0.8008 (15)	1.0207 (6)	0.94351 (13)	0.094 (3)	
H1L	0.7316	0.9756	0.9333	0.113*	0.50
H1LA	0.7639	0.9740	0.9318	0.113*	0.50
Cl1	0.647 (2)	1.0539 (12)	0.96442 (17)	0.140 (4)	0.50
Cl1A	0.569 (3)	1.0590 (13)	0.95422 (18)	0.156 (5)	0.50
Cl2	0.9810 (6)	0.9642 (2)	0.95867 (5)	0.1786 (16)	
Cl3	0.8989 (7)	1.1142 (2)	0.92953 (5)	0.1788 (16)	
O2L	0.5211 (11)	0.7631 (6)	0.82855 (13)	0.168 (3)	
H2L	0.3996	0.7479	0.8273	0.252*	
C3L	0.5371 (17)	0.8457 (10)	0.8417 (2)	0.171 (6)	
H3LA	0.6794	0.8641	0.8437	0.205*	
H3LB	0.4755	0.8354	0.8564	0.205*	
C4L	0.4265 (19)	0.9186 (9)	0.82927 (17)	0.159 (5)	
H4LB	0.4507	0.9098	0.8135	0.238*	
H4LC	0.4730	0.9817	0.8337	0.238*	
H4LD	0.2824	0.9127	0.8323	0.238*	

### Atomic displacement parameters (Å^2^)

**Table:**

	*U*^11^	*U*^22^	*U*^33^	*U*^12^	*U*^13^	*U*^23^
C1	0.046 (5)	0.044 (4)	0.050 (4)	−0.003 (4)	0.004 (4)	−0.000 (4)
N2	0.062 (4)	0.050 (4)	0.059 (4)	−0.012 (4)	0.005 (3)	−0.013 (3)
N3	0.057 (4)	0.050 (4)	0.061 (4)	−0.015 (4)	0.011 (3)	−0.008 (3)
C4	0.050 (6)	0.050 (5)	0.045 (4)	−0.004 (4)	−0.001 (4)	0.010 (4)
N5	0.047 (4)	0.036 (3)	0.047 (3)	−0.016 (3)	−0.006 (3)	−0.003 (3)
C6	0.042 (5)	0.048 (4)	0.054 (4)	−0.005 (4)	−0.009 (4)	0.014 (4)
N7	0.052 (4)	0.050 (4)	0.050 (3)	−0.006 (4)	−0.001 (3)	−0.000 (3)
N8	0.065 (5)	0.055 (4)	0.050 (3)	−0.015 (4)	0.000 (4)	0.002 (3)
C9	0.059 (6)	0.041 (4)	0.041 (4)	−0.007 (4)	−0.001 (4)	0.002 (3)
N10	0.048 (4)	0.038 (4)	0.048 (3)	−0.002 (3)	0.010 (3)	−0.005 (3)
N12	0.056 (5)	0.057 (4)	0.057 (4)	−0.007 (4)	0.004 (3)	−0.004 (3)
N13	0.059 (5)	0.050 (4)	0.055 (4)	−0.009 (4)	0.006 (3)	−0.006 (3)
C11	0.061 (6)	0.040 (4)	0.046 (4)	−0.010 (4)	−0.004 (4)	0.008 (4)
C14	0.055 (5)	0.035 (4)	0.053 (4)	−0.009 (4)	−0.000 (4)	−0.002 (3)
N15	0.048 (4)	0.043 (4)	0.045 (3)	−0.003 (3)	−0.000 (3)	−0.010 (3)
C16	0.054 (6)	0.043 (5)	0.054 (4)	−0.009 (5)	0.007 (4)	−0.007 (4)
C17	0.073 (6)	0.073 (6)	0.090 (6)	−0.006 (6)	−0.014 (5)	0.021 (5)
C18	0.070 (6)	0.065 (6)	0.076 (5)	−0.027 (6)	−0.019 (5)	0.027 (4)
C19	0.056 (6)	0.048 (5)	0.069 (5)	0.001 (5)	0.005 (5)	0.003 (4)
C20	0.042 (5)	0.061 (5)	0.097 (6)	−0.011 (5)	−0.019 (4)	0.016 (5)
C21	0.061 (6)	0.053 (5)	0.089 (6)	0.001 (5)	−0.005 (5)	0.022 (4)
O22	0.052 (3)	0.041 (3)	0.084 (3)	−0.008 (3)	−0.006 (3)	0.005 (3)
C23	0.057 (6)	0.040 (4)	0.080 (5)	0.000 (4)	−0.003 (5)	0.004 (4)
C24	0.050 (6)	0.047 (5)	0.108 (7)	−0.000 (4)	−0.012 (5)	−0.002 (5)
C25	0.074 (7)	0.056 (5)	0.091 (6)	−0.003 (6)	−0.019 (6)	−0.005 (5)
C26	0.091 (8)	0.078 (7)	0.130 (9)	−0.019 (7)	−0.023 (7)	−0.010 (6)
C27	0.061 (6)	0.064 (6)	0.128 (8)	−0.023 (5)	−0.008 (6)	0.020 (6)
C28	0.060 (6)	0.048 (5)	0.110 (7)	−0.007 (5)	−0.001 (6)	0.022 (5)
C29	0.133 (9)	0.098 (7)	0.098 (7)	−0.004 (7)	−0.006 (7)	−0.042 (6)
C30	0.066 (7)	0.069 (6)	0.121 (8)	0.001 (6)	0.003 (6)	0.017 (6)
C31	0.129 (10)	0.127 (9)	0.098 (7)	0.005 (8)	0.018 (8)	0.024 (6)
C32	0.093 (8)	0.108 (8)	0.180 (10)	−0.010 (7)	0.042 (7)	0.057 (7)
C33	0.054 (5)	0.051 (5)	0.048 (4)	−0.009 (4)	−0.010 (4)	0.003 (4)
C34	0.062 (6)	0.106 (7)	0.051 (4)	−0.040 (5)	0.007 (4)	0.001 (5)
C35	0.055 (6)	0.115 (7)	0.048 (4)	−0.041 (6)	0.000 (4)	−0.018 (4)
C36	0.067 (6)	0.052 (5)	0.045 (4)	−0.003 (5)	−0.006 (4)	−0.002 (4)
C37	0.036 (4)	0.044 (4)	0.066 (5)	−0.011 (4)	−0.004 (4)	0.004 (4)
C38	0.058 (5)	0.039 (4)	0.047 (4)	−0.019 (4)	−0.007 (4)	0.000 (3)
O39	0.044 (3)	0.077 (4)	0.051 (3)	−0.022 (3)	0.007 (3)	−0.006 (3)
C40	0.054 (5)	0.077 (6)	0.043 (4)	−0.018 (5)	0.002 (4)	0.003 (4)
C41	0.063 (6)	0.076 (6)	0.055 (5)	−0.023 (5)	0.000 (4)	0.001 (4)
C42	0.071 (7)	0.071 (6)	0.058 (5)	−0.020 (6)	−0.000 (5)	0.001 (4)
C43	0.060 (6)	0.078 (6)	0.067 (5)	0.008 (5)	−0.008 (5)	−0.006 (4)
C44	0.039 (5)	0.084 (6)	0.064 (5)	−0.006 (5)	−0.002 (4)	0.000 (4)
C45	0.066 (6)	0.059 (5)	0.050 (4)	−0.008 (5)	−0.008 (4)	0.005 (4)
C46	0.140 (9)	0.077 (6)	0.074 (5)	0.001 (7)	0.012 (6)	−0.003 (5)
C47	0.061 (6)	0.084 (7)	0.061 (5)	−0.014 (6)	0.005 (5)	0.014 (5)
C48	0.100 (8)	0.077 (6)	0.098 (6)	−0.013 (6)	0.014 (6)	0.020 (5)
C49	0.064 (7)	0.121 (8)	0.100 (6)	0.006 (6)	0.004 (5)	0.014 (6)
C50	0.058 (6)	0.042 (4)	0.045 (4)	−0.002 (4)	0.002 (4)	−0.005 (3)
C51	0.053 (6)	0.064 (5)	0.050 (4)	0.008 (5)	0.005 (4)	0.001 (4)
C52	0.072 (6)	0.063 (5)	0.048 (4)	0.006 (5)	−0.009 (4)	−0.006 (4)
C53	0.068 (6)	0.057 (5)	0.049 (4)	0.004 (5)	−0.013 (5)	−0.015 (4)
C54	0.065 (6)	0.049 (4)	0.048 (4)	0.015 (5)	−0.003 (4)	−0.013 (4)
C55	0.056 (6)	0.063 (5)	0.051 (4)	−0.008 (5)	−0.001 (4)	0.006 (4)
O56	0.075 (4)	0.084 (4)	0.071 (3)	0.020 (4)	−0.016 (3)	−0.034 (3)
C57	0.081 (7)	0.076 (6)	0.070 (5)	0.026 (6)	−0.015 (5)	−0.028 (5)
C58	0.125 (10)	0.090 (7)	0.075 (6)	0.001 (8)	−0.018 (6)	−0.020 (6)
C59	0.150 (12)	0.119 (9)	0.101 (8)	−0.034 (10)	0.024 (8)	−0.050 (7)
C60	0.117 (10)	0.175 (12)	0.145 (11)	−0.022 (10)	0.022 (9)	−0.075 (10)
C61	0.078 (8)	0.105 (9)	0.133 (9)	0.023 (7)	−0.021 (7)	−0.078 (7)
C62	0.067 (7)	0.099 (8)	0.082 (6)	0.012 (6)	−0.020 (5)	−0.045 (6)
C63	0.141 (18)	0.139 (16)	0.113 (14)	−0.047 (13)	0.034 (13)	−0.021 (11)
C63A	0.15 (3)	0.18 (3)	0.16 (2)	−0.012 (18)	0.014 (18)	−0.003 (18)
C64	0.114 (9)	0.078 (7)	0.076 (6)	−0.002 (7)	−0.037 (6)	−0.016 (5)
C65	0.118 (10)	0.176 (12)	0.139 (9)	0.018 (10)	0.041 (8)	0.019 (8)
C66	0.189 (13)	0.088 (7)	0.154 (9)	−0.020 (9)	−0.040 (9)	−0.047 (7)
C1L	0.148 (10)	0.076 (6)	0.059 (5)	0.011 (7)	−0.006 (6)	0.004 (5)
Cl1	0.182 (11)	0.114 (6)	0.124 (8)	−0.015 (7)	0.046 (7)	−0.032 (7)
Cl1A	0.221 (15)	0.121 (6)	0.126 (9)	0.005 (9)	0.050 (8)	−0.009 (8)
Cl2	0.229 (4)	0.172 (3)	0.135 (2)	−0.016 (3)	−0.078 (3)	0.038 (2)
Cl3	0.278 (5)	0.113 (2)	0.145 (2)	0.014 (3)	0.074 (3)	0.048 (2)
O2L	0.093 (6)	0.203 (9)	0.208 (8)	0.003 (7)	0.045 (6)	0.001 (7)
C3L	0.086 (9)	0.165 (11)	0.261 (13)	0.054 (8)	−0.082 (9)	−0.100 (10)
C4L	0.130 (11)	0.202 (13)	0.144 (10)	−0.021 (12)	0.033 (9)	0.007 (9)

### Geometric parameters (Å, º)

**Table:**

C1—N2	1.317 (8)	C42—H42	1.0000
C1—N15	1.381 (7)	C43—C44	1.541 (8)
C1—C16	1.456 (9)	C43—H43A	0.9900
N2—N3	1.413 (7)	C43—H43B	0.9900
N3—C4	1.286 (7)	C44—C45	1.521 (9)
C4—N5	1.370 (8)	C44—H44A	0.9900
C4—N15	1.387 (8)	C44—H44B	0.9900
N5—C9	1.369 (7)	C45—C47	1.527 (9)
N5—C6	1.413 (8)	C45—H45	1.0000
C6—N7	1.293 (7)	C46—H46A	0.9800
C6—C33	1.472 (9)	C46—H46B	0.9800
N7—N8	1.398 (7)	C46—H46C	0.9800
N8—C9	1.287 (8)	C47—C48	1.546 (9)
C9—N10	1.388 (8)	C47—C49	1.549 (10)
N10—C14	1.374 (7)	C47—H47	1.0000
N10—C11	1.398 (8)	C48—H48A	0.9800
N12—C11	1.291 (8)	C48—H48B	0.9800
N12—N13	1.415 (7)	C48—H48C	0.9800
N13—C14	1.292 (8)	C49—H49A	0.9800
C11—C50	1.471 (8)	C49—H49B	0.9800
C14—N15	1.372 (8)	C49—H49C	0.9800
C16—C17	1.373 (9)	C50—C51	1.364 (9)
C16—C21	1.373 (9)	C50—C55	1.378 (9)
C17—C18	1.368 (9)	C51—C52	1.388 (8)
C17—H17	0.9500	C51—H51	0.9500
C18—C19	1.369 (9)	C52—C53	1.370 (9)
C18—H18	0.9500	C52—H52	0.9500
C19—C20	1.358 (9)	C53—O56	1.382 (7)
C19—O22	1.369 (8)	C53—C54	1.390 (9)
C20—C21	1.398 (9)	C54—C55	1.386 (8)
C20—H20	0.9500	C54—H54	0.9500
C21—H21	0.9500	C55—H55	0.9500
O22—C23	1.466 (7)	O56—C57	1.461 (8)
C23—C24	1.494 (9)	C57—C58	1.508 (10)
C23—C28	1.526 (9)	C57—C62	1.523 (10)
C23—H23	1.0000	C57—H57	1.0000
C24—C25	1.514 (9)	C58—C59	1.521 (13)
C24—H24A	0.9900	C58—H58A	0.9900
C24—H24B	0.9900	C58—H58B	0.9900
C25—C26	1.507 (10)	C59—C60	1.523 (12)
C25—C29	1.522 (10)	C59—C63	1.533 (15)
C25—H25	1.0000	C59—C63A	1.569 (19)
C26—C27	1.514 (10)	C59—H59	1.0000
C26—H26A	0.9900	C59—H59A	1.0000
C26—H26B	0.9900	C60—C61	1.511 (12)
C27—C28	1.547 (10)	C60—H60A	0.9900
C27—H27A	0.9900	C60—H60B	0.9900
C27—H27B	0.9900	C61—C62	1.537 (11)
C28—C30	1.520 (10)	C61—H61A	0.9900
C28—H28	1.0000	C61—H61B	0.9900
C29—H29A	0.9800	C62—C64	1.540 (11)
C29—H29B	0.9800	C62—H62	1.0000
C29—H29C	0.9800	C63—H63A	0.9800
C30—C31	1.517 (10)	C63—H63B	0.9800
C30—C32	1.588 (11)	C63—H63C	0.9800
C30—H30	1.0000	C63A—H63D	0.9800
C31—H31A	0.9800	C63A—H63E	0.9800
C31—H31B	0.9800	C63A—H63F	0.9800
C31—H31C	0.9800	C64—C65	1.502 (11)
C32—H32A	0.9800	C64—C66	1.524 (10)
C32—H32B	0.9800	C64—H64	1.0000
C32—H32C	0.9800	C65—H65A	0.9800
C33—C34	1.374 (8)	C65—H65B	0.9800
C33—C38	1.382 (8)	C65—H65C	0.9800
C34—C35	1.396 (9)	C66—H66A	0.9800
C34—H34	0.9500	C66—H66B	0.9800
C35—C36	1.383 (9)	C66—H66C	0.9800
C35—H35	0.9500	C1L—Cl3	1.698 (8)
C36—O39	1.350 (8)	C1L—Cl1	1.699 (18)
C36—C37	1.387 (8)	C1L—Cl2	1.710 (9)
C37—C38	1.378 (8)	C1L—Cl1A	1.76 (2)
C37—H37	0.9500	C1L—H1L	1.0000
C38—H38	0.9500	C1L—H1LA	1.0000
O39—C40	1.456 (7)	O2L—C3L	1.418 (11)
C40—C41	1.498 (9)	O2L—H2L	0.8400
C40—C45	1.538 (9)	C3L—C4L	1.474 (13)
C40—H40	1.0000	C3L—H3LA	0.9900
C41—C42	1.530 (10)	C3L—H3LB	0.9900
C41—H41A	0.9900	C4L—H4LB	0.9800
C41—H41B	0.9900	C4L—H4LC	0.9800
C42—C46	1.507 (9)	C4L—H4LD	0.9800
C42—C43	1.536 (9)		
			
N2—C1—N15	107.9 (6)	C44—C43—H43B	109.3
N2—C1—C16	126.0 (7)	H43A—C43—H43B	108.0
N15—C1—C16	125.4 (6)	C45—C44—C43	111.3 (7)
C1—N2—N3	109.0 (5)	C45—C44—H44A	109.4
C4—N3—N2	106.5 (6)	C43—C44—H44A	109.4
N3—C4—N5	133.0 (7)	C45—C44—H44B	109.4
N3—C4—N15	110.8 (6)	C43—C44—H44B	109.4
N5—C4—N15	116.2 (6)	H44A—C44—H44B	108.0
C9—N5—C4	123.1 (6)	C44—C45—C47	112.7 (7)
C9—N5—C6	101.8 (5)	C44—C45—C40	111.5 (6)
C4—N5—C6	134.8 (6)	C47—C45—C40	112.2 (6)
N7—C6—N5	109.5 (6)	C44—C45—H45	106.6
N7—C6—C33	121.4 (7)	C47—C45—H45	106.6
N5—C6—C33	129.2 (7)	C40—C45—H45	106.6
C6—N7—N8	109.5 (6)	C42—C46—H46A	109.5
C9—N8—N7	104.9 (6)	C42—C46—H46B	109.5
N8—C9—N5	114.3 (7)	H46A—C46—H46B	109.5
N8—C9—N10	127.9 (7)	C42—C46—H46C	109.5
N5—C9—N10	117.7 (6)	H46A—C46—H46C	109.5
C14—N10—C9	122.1 (6)	H46B—C46—H46C	109.5
C14—N10—C11	104.3 (6)	C45—C47—C48	112.0 (6)
C9—N10—C11	133.3 (6)	C45—C47—C49	113.7 (7)
C11—N12—N13	110.3 (6)	C48—C47—C49	108.2 (7)
C14—N13—N12	104.6 (6)	C45—C47—H47	107.5
N12—C11—N10	108.2 (6)	C48—C47—H47	107.5
N12—C11—C50	126.2 (7)	C49—C47—H47	107.5
N10—C11—C50	125.3 (7)	C47—C48—H48A	109.5
N13—C14—N15	130.6 (7)	C47—C48—H48B	109.5
N13—C14—N10	112.6 (6)	H48A—C48—H48B	109.5
N15—C14—N10	116.8 (7)	C47—C48—H48C	109.5
C14—N15—C1	130.5 (6)	H48A—C48—H48C	109.5
C14—N15—C4	123.8 (6)	H48B—C48—H48C	109.5
C1—N15—C4	105.7 (6)	C47—C49—H49A	109.5
C17—C16—C21	118.9 (8)	C47—C49—H49B	109.5
C17—C16—C1	119.5 (8)	H49A—C49—H49B	109.5
C21—C16—C1	121.6 (7)	C47—C49—H49C	109.5
C18—C17—C16	121.4 (8)	H49A—C49—H49C	109.5
C18—C17—H17	119.3	H49B—C49—H49C	109.5
C16—C17—H17	119.3	C51—C50—C55	120.5 (7)
C17—C18—C19	120.1 (8)	C51—C50—C11	120.7 (7)
C17—C18—H18	120.0	C55—C50—C11	118.6 (7)
C19—C18—H18	120.0	C50—C51—C52	120.8 (7)
C20—C19—O22	115.7 (8)	C50—C51—H51	119.6
C20—C19—C18	119.3 (8)	C52—C51—H51	119.6
O22—C19—C18	124.9 (7)	C53—C52—C51	118.6 (7)
C19—C20—C21	121.0 (8)	C53—C52—H52	120.7
C19—C20—H20	119.5	C51—C52—H52	120.7
C21—C20—H20	119.5	C52—C53—O56	124.1 (7)
C16—C21—C20	119.3 (7)	C52—C53—C54	121.4 (6)
C16—C21—H21	120.4	O56—C53—C54	114.5 (7)
C20—C21—H21	120.4	C55—C54—C53	118.9 (7)
C19—O22—C23	117.7 (6)	C55—C54—H54	120.5
O22—C23—C24	110.2 (6)	C53—C54—H54	120.5
O22—C23—C28	105.1 (6)	C50—C55—C54	119.8 (7)
C24—C23—C28	112.7 (6)	C50—C55—H55	120.1
O22—C23—H23	109.6	C54—C55—H55	120.1
C24—C23—H23	109.6	C53—O56—C57	118.0 (6)
C28—C23—H23	109.6	O56—C57—C58	110.2 (8)
C23—C24—C25	114.2 (7)	O56—C57—C62	105.2 (6)
C23—C24—H24A	108.7	C58—C57—C62	112.6 (7)
C25—C24—H24A	108.7	O56—C57—H57	109.6
C23—C24—H24B	108.7	C58—C57—H57	109.6
C25—C24—H24B	108.7	C62—C57—H57	109.6
H24A—C24—H24B	107.6	C57—C58—C59	113.9 (9)
C26—C25—C24	108.2 (7)	C57—C58—H58A	108.8
C26—C25—C29	110.2 (7)	C59—C58—H58A	108.8
C24—C25—C29	111.7 (7)	C57—C58—H58B	108.8
C26—C25—H25	108.9	C59—C58—H58B	108.8
C24—C25—H25	108.9	H58A—C58—H58B	107.7
C29—C25—H25	108.9	C58—C59—C60	108.5 (10)
C25—C26—C27	111.2 (7)	C58—C59—C63	118.3 (16)
C25—C26—H26A	109.4	C60—C59—C63	109.3 (14)
C27—C26—H26A	109.4	C58—C59—C63A	93.5 (19)
C25—C26—H26B	109.4	C60—C59—C63A	112 (2)
C27—C26—H26B	109.4	C58—C59—H59	106.7
H26A—C26—H26B	108.0	C60—C59—H59	106.7
C26—C27—C28	113.9 (8)	C63—C59—H59	106.7
C26—C27—H27A	108.8	C63A—C59—H59	127.5
C28—C27—H27A	108.8	C58—C59—H59A	113.7
C26—C27—H27B	108.8	C60—C59—H59A	113.7
C28—C27—H27B	108.8	C63—C59—H59A	92.6
H27A—C27—H27B	107.7	C63A—C59—H59A	113.7
C30—C28—C23	112.3 (7)	C61—C60—C59	113.8 (9)
C30—C28—C27	115.3 (8)	C61—C60—H60A	108.8
C23—C28—C27	108.0 (6)	C59—C60—H60A	108.8
C30—C28—H28	106.9	C61—C60—H60B	108.8
C23—C28—H28	106.9	C59—C60—H60B	108.8
C27—C28—H28	106.9	H60A—C60—H60B	107.7
C25—C29—H29A	109.5	C60—C61—C62	112.4 (9)
C25—C29—H29B	109.5	C60—C61—H61A	109.1
H29A—C29—H29B	109.5	C62—C61—H61A	109.1
C25—C29—H29C	109.5	C60—C61—H61B	109.1
H29A—C29—H29C	109.5	C62—C61—H61B	109.1
H29B—C29—H29C	109.5	H61A—C61—H61B	107.9
C31—C30—C28	113.7 (8)	C57—C62—C61	109.6 (8)
C31—C30—C32	108.7 (8)	C57—C62—C64	113.6 (7)
C28—C30—C32	109.4 (7)	C61—C62—C64	112.9 (9)
C31—C30—H30	108.3	C57—C62—H62	106.8
C28—C30—H30	108.3	C61—C62—H62	106.8
C32—C30—H30	108.3	C64—C62—H62	106.8
C30—C31—H31A	109.5	C59—C63—H63A	109.5
C30—C31—H31B	109.5	C59—C63—H63B	109.5
H31A—C31—H31B	109.5	C59—C63—H63C	109.5
C30—C31—H31C	109.5	C59—C63A—H63D	109.5
H31A—C31—H31C	109.5	C59—C63A—H63E	109.5
H31B—C31—H31C	109.5	H63D—C63A—H63E	109.5
C30—C32—H32A	109.5	C59—C63A—H63F	109.5
C30—C32—H32B	109.5	H63D—C63A—H63F	109.5
H32A—C32—H32B	109.5	H63E—C63A—H63F	109.5
C30—C32—H32C	109.5	C65—C64—C66	113.2 (10)
H32A—C32—H32C	109.5	C65—C64—C62	110.7 (8)
H32B—C32—H32C	109.5	C66—C64—C62	110.9 (8)
C34—C33—C38	118.3 (7)	C65—C64—H64	107.3
C34—C33—C6	122.5 (7)	C66—C64—H64	107.3
C38—C33—C6	119.1 (6)	C62—C64—H64	107.3
C33—C34—C35	121.3 (7)	C64—C65—H65A	109.5
C33—C34—H34	119.3	C64—C65—H65B	109.5
C35—C34—H34	119.3	H65A—C65—H65B	109.5
C36—C35—C34	120.3 (7)	C64—C65—H65C	109.5
C36—C35—H35	119.8	H65A—C65—H65C	109.5
C34—C35—H35	119.8	H65B—C65—H65C	109.5
O39—C36—C35	125.5 (6)	C64—C66—H66A	109.5
O39—C36—C37	116.8 (7)	C64—C66—H66B	109.5
C35—C36—C37	117.6 (7)	H66A—C66—H66B	109.5
C38—C37—C36	121.9 (7)	C64—C66—H66C	109.5
C38—C37—H37	119.0	H66A—C66—H66C	109.5
C36—C37—H37	119.0	H66B—C66—H66C	109.5
C37—C38—C33	120.4 (6)	Cl3—C1L—Cl1	113.1 (8)
C37—C38—H38	119.8	Cl3—C1L—Cl2	111.2 (6)
C33—C38—H38	119.8	Cl1—C1L—Cl2	98.6 (6)
C36—O39—C40	118.1 (5)	Cl3—C1L—Cl1A	106.5 (7)
O39—C40—C41	110.0 (6)	Cl2—C1L—Cl1A	124.0 (6)
O39—C40—C45	106.7 (6)	Cl3—C1L—H1L	111.1
C41—C40—C45	113.4 (6)	Cl1—C1L—H1L	111.1
O39—C40—H40	108.9	Cl2—C1L—H1L	111.1
C41—C40—H40	108.9	Cl1A—C1L—H1L	91.2
C45—C40—H40	108.9	Cl3—C1L—H1LA	104.4
C40—C41—C42	114.5 (7)	Cl1—C1L—H1LA	124.4
C40—C41—H41A	108.6	Cl2—C1L—H1LA	104.4
C42—C41—H41A	108.6	Cl1A—C1L—H1LA	104.4
C40—C41—H41B	108.6	C3L—O2L—H2L	109.5
C42—C41—H41B	108.6	O2L—C3L—C4L	104.2 (10)
H41A—C41—H41B	107.6	O2L—C3L—H3LA	110.9
C46—C42—C41	113.2 (8)	C4L—C3L—H3LA	110.9
C46—C42—C43	112.4 (7)	O2L—C3L—H3LB	110.9
C41—C42—C43	108.3 (6)	C4L—C3L—H3LB	110.9
C46—C42—H42	107.6	H3LA—C3L—H3LB	108.9
C41—C42—H42	107.6	C3L—C4L—H4LB	109.5
C43—C42—H42	107.6	C3L—C4L—H4LC	109.5
C42—C43—C44	111.4 (6)	H4LB—C4L—H4LC	109.5
C42—C43—H43A	109.3	C3L—C4L—H4LD	109.5
C44—C43—H43A	109.3	H4LB—C4L—H4LD	109.5
C42—C43—H43B	109.3	H4LC—C4L—H4LD	109.5
			
N15—C1—N2—N3	−1.0 (7)	C24—C23—C28—C27	51.1 (8)
C16—C1—N2—N3	170.1 (7)	C26—C27—C28—C30	−179.2 (7)
C1—N2—N3—C4	−0.8 (8)	C26—C27—C28—C23	−52.7 (9)
N2—N3—C4—N5	−177.7 (7)	C23—C28—C30—C31	52.9 (9)
N2—N3—C4—N15	2.2 (7)	C27—C28—C30—C31	177.3 (7)
N3—C4—N5—C9	176.0 (7)	C23—C28—C30—C32	174.7 (6)
N15—C4—N5—C9	−3.9 (9)	C27—C28—C30—C32	−61.0 (9)
N3—C4—N5—C6	2.1 (13)	N7—C6—C33—C34	159.3 (7)
N15—C4—N5—C6	−177.8 (6)	N5—C6—C33—C34	−19.8 (11)
C9—N5—C6—N7	1.2 (7)	N7—C6—C33—C38	−17.2 (10)
C4—N5—C6—N7	176.0 (6)	N5—C6—C33—C38	163.7 (6)
C9—N5—C6—C33	−179.6 (6)	C38—C33—C34—C35	−2.4 (11)
C4—N5—C6—C33	−4.8 (11)	C6—C33—C34—C35	−179.0 (7)
N5—C6—N7—N8	−0.6 (7)	C33—C34—C35—C36	3.6 (12)
C33—C6—N7—N8	−179.9 (6)	C34—C35—C36—O39	173.5 (7)
C6—N7—N8—C9	−0.2 (7)	C34—C35—C36—C37	−2.0 (12)
N7—N8—C9—N5	1.1 (8)	O39—C36—C37—C38	−176.4 (6)
N7—N8—C9—N10	179.3 (6)	C35—C36—C37—C38	−0.5 (10)
C4—N5—C9—N8	−177.1 (6)	C36—C37—C38—C33	1.6 (10)
C6—N5—C9—N8	−1.5 (7)	C34—C33—C38—C37	−0.1 (10)
C4—N5—C9—N10	4.6 (9)	C6—C33—C38—C37	176.6 (6)
C6—N5—C9—N10	−179.8 (5)	C35—C36—O39—C40	−7.4 (11)
N8—C9—N10—C14	−178.0 (7)	C37—C36—O39—C40	168.2 (6)
N5—C9—N10—C14	0.1 (9)	C36—O39—C40—C41	87.0 (8)
N8—C9—N10—C11	−6.1 (12)	C36—O39—C40—C45	−149.7 (6)
N5—C9—N10—C11	172.0 (6)	O39—C40—C41—C42	68.3 (8)
C11—N12—N13—C14	−0.8 (8)	C45—C40—C41—C42	−51.0 (8)
N13—N12—C11—N10	0.7 (8)	C40—C41—C42—C46	179.6 (6)
N13—N12—C11—C50	176.1 (6)	C40—C41—C42—C43	54.4 (8)
C14—N10—C11—N12	−0.3 (7)	C46—C42—C43—C44	176.8 (7)
C9—N10—C11—N12	−173.2 (6)	C41—C42—C43—C44	−57.5 (8)
C14—N10—C11—C50	−175.7 (6)	C42—C43—C44—C45	58.5 (8)
C9—N10—C11—C50	11.3 (11)	C43—C44—C45—C47	−179.7 (5)
N12—N13—C14—N15	179.1 (6)	C43—C44—C45—C40	−52.5 (8)
N12—N13—C14—N10	0.7 (7)	O39—C40—C45—C44	−72.3 (7)
C9—N10—C14—N13	173.6 (6)	C41—C40—C45—C44	48.9 (8)
C11—N10—C14—N13	−0.3 (7)	O39—C40—C45—C47	55.3 (8)
C9—N10—C14—N15	−5.1 (9)	C41—C40—C45—C47	176.5 (6)
C11—N10—C14—N15	−179.0 (5)	C44—C45—C47—C48	−59.1 (8)
N13—C14—N15—C1	9.1 (12)	C40—C45—C47—C48	174.1 (6)
N10—C14—N15—C1	−172.5 (6)	C44—C45—C47—C49	177.9 (6)
N13—C14—N15—C4	−172.5 (7)	C40—C45—C47—C49	51.0 (9)
N10—C14—N15—C4	5.9 (9)	N12—C11—C50—C51	−124.7 (8)
N2—C1—N15—C14	−179.2 (6)	N10—C11—C50—C51	49.9 (10)
C16—C1—N15—C14	9.7 (11)	N12—C11—C50—C55	50.6 (10)
N2—C1—N15—C4	2.2 (7)	N10—C11—C50—C55	−134.7 (7)
C16—C1—N15—C4	−168.9 (7)	C55—C50—C51—C52	−1.0 (10)
N3—C4—N15—C14	178.5 (6)	C11—C50—C51—C52	174.3 (6)
N5—C4—N15—C14	−1.6 (9)	C50—C51—C52—C53	0.6 (11)
N3—C4—N15—C1	−2.8 (7)	C51—C52—C53—O56	179.3 (6)
N5—C4—N15—C1	177.1 (5)	C51—C52—C53—C54	0.7 (11)
N2—C1—C16—C17	−80.2 (9)	C52—C53—C54—C55	−1.6 (11)
N15—C1—C16—C17	89.3 (9)	O56—C53—C54—C55	179.7 (6)
N2—C1—C16—C21	99.0 (9)	C51—C50—C55—C54	0.0 (10)
N15—C1—C16—C21	−91.5 (9)	C11—C50—C55—C54	−175.3 (6)
C21—C16—C17—C18	0.7 (12)	C53—C54—C55—C50	1.2 (10)
C1—C16—C17—C18	180.0 (7)	C52—C53—O56—C57	20.5 (11)
C16—C17—C18—C19	0.1 (12)	C54—C53—O56—C57	−160.8 (7)
C17—C18—C19—C20	−1.0 (12)	C53—O56—C57—C58	76.7 (8)
C17—C18—C19—O22	177.0 (7)	C53—O56—C57—C62	−161.7 (7)
O22—C19—C20—C21	−177.0 (6)	O56—C57—C58—C59	61.5 (9)
C18—C19—C20—C21	1.1 (12)	C62—C57—C58—C59	−55.7 (10)
C17—C16—C21—C20	−0.6 (11)	C57—C58—C59—C60	53.6 (11)
C1—C16—C21—C20	−179.8 (7)	C57—C58—C59—C63	178.8 (14)
C19—C20—C21—C16	−0.3 (12)	C57—C58—C59—C63A	168 (2)
C20—C19—O22—C23	−153.4 (6)	C58—C59—C60—C61	−53.4 (13)
C18—C19—O22—C23	28.6 (10)	C63—C59—C60—C61	176.3 (15)
C19—O22—C23—C24	66.4 (8)	C63A—C59—C60—C61	−155 (2)
C19—O22—C23—C28	−172.0 (6)	C59—C60—C61—C62	55.0 (13)
O22—C23—C24—C25	60.7 (8)	O56—C57—C62—C61	−67.1 (9)
C28—C23—C24—C25	−56.3 (8)	C58—C57—C62—C61	52.9 (10)
C23—C24—C25—C26	56.9 (9)	O56—C57—C62—C64	60.2 (9)
C23—C24—C25—C29	178.3 (7)	C58—C57—C62—C64	−179.7 (8)
C24—C25—C26—C27	−56.0 (9)	C60—C61—C62—C57	−52.6 (10)
C29—C25—C26—C27	−178.4 (7)	C60—C61—C62—C64	179.7 (8)
C25—C26—C27—C28	57.3 (9)	C57—C62—C64—C65	53.1 (10)
O22—C23—C28—C30	59.3 (8)	C61—C62—C64—C65	178.7 (7)
C24—C23—C28—C30	179.3 (7)	C57—C62—C64—C66	179.5 (8)
O22—C23—C28—C27	−68.9 (8)	C61—C62—C64—C66	−54.9 (10)

### Hydrogen-bond geometry (Å, º)

**Table:**

*D*—H···*A*	*D*—H	H···*A*	*D*···*A*	*D*—H···*A*
O2*L*—H2*L*···N12^i^	0.84	2.11	2.896 (10)	156

Symmetry code: (i) *x*−1, *y*, *z*.
